# Activated Biocarbons Based on *Salvia officinalis* L. Processing Residue as Adsorbents of Pollutants from Drinking Water

**DOI:** 10.3390/molecules30143037

**Published:** 2025-07-19

**Authors:** Joanna Koczenasz, Piotr Nowicki, Karina Tokarska, Małgorzata Wiśniewska

**Affiliations:** 1 Department of Applied Chemistry, Faculty of Chemistry, Adam Mickiewicz University in Poznań, Uniwersytetu Poznańskiego 8, 61-614 Poznań, Poland; joakoc6@st.amu.edu.pl; 2 Department of Radiochemistry and Environmental Chemistry, Institute of Chemical Sciences, Faculty of Chemistry, Maria Curie-Sklodowska University in Lublin, M. Curie-Sklodowska Sq. 3, 20-031 Lublin, Poland; karina.tokarska@mail.umcs.pl

**Keywords:** sage stems, waste biomass utilization, activated biocarbons, chemical activation, microwave and conventional heating, adsorption

## Abstract

This study presents research on the production of activated biocarbons derived from herbal waste. Sage stems were chemically activated with two activating agents of different chemical natures—H_3_PO_4_ and K_2_CO_3_—and subjected to two thermal treatment methods: conventional and microwave heating. The effect of the activating agent type and heating method on the basic physicochemical properties of the resulting activated biocarbons was investigated. These properties included surface morphology, elemental composition, ash content, pH of aqueous extracts, the content and nature of surface functional groups, points of zero charge, and isoelectric points, as well as the type of porous structure formed. In addition, the potential of the prepared carbonaceous materials as adsorbents of model organic (represented by Triton X-100 and methylene blue) and inorganic (represented by iodine) pollutants was assessed. The influence of the initial adsorbate concentration (5–150 (dye) and 10–800 mg/dm^3^ (surfactant)), temperature (20–40 °C), and pH (2–10) of the system on the efficiency of contaminant removal from aqueous solutions was evaluated. The adsorption kinetics were also investigated to better understand the rate and mechanism of contaminant uptake by the prepared activated biocarbons. The results showed that materials activated with orthophosphoric acid exhibited a significantly higher sorption capacity for all tested adsorbates compared to their potassium carbonate-activated counterparts. Microwave heating was found to be more effective in promoting the formation of a well-developed specific surface area (471–1151 m^2^/g) and porous structure (mean pore size 2.17–3.84 nm), which directly enhanced the sorption capacity of both organic and inorganic contaminants. The maximum adsorption capacities for iodine, methylene blue, and Triton X-100 reached the levels of 927.0, 298.4, and 644.3 mg/g, respectively, on the surface of the H_3_PO_4_-activated sample obtained by microwave heating. It was confirmed that the heating method used during the activation step plays a key role in determining the physicochemical properties and sorption efficiency of activated biocarbons.

## 1. Introduction

Searching for new purification methods still remains one of the main challenges faced by modern scientists. To meet the high demand for clean drinking water and solve problems related to environment pollution, scientists are looking for innovative water remediation strategies that are not only effective, but also ecological. One of the solutions that meets the above requirements is the use of activated biocarbons as adsorbents of pollutants present in water.

Activated carbons are produced by exposing organic precursors to high-temperature treatment in the presence of appropriate activating agents. The choice of the activation method has a significant impact on the properties of the resulting materials [1,2]. Two main types of activation procedure are distinguished: physical activation (also called thermal activation), which involves the use of gases such as steam or carbon dioxide at temperatures ranging from 800 to 1000 °C, and chemical activation, which consists of mixing the precursor with a solution of potassium hydroxide, zinc chloride, or orthophosphoric acid, followed by thermal treatment at temperatures ranging from 500 to 800 °C.

The main advantages of the chemical activation method in comparison to the physical one include lower temperature requirements, larger specific surface area, and more developed porous structure, as well as shorter processing time and higher mass yield of the activated carbon [3,4]. The activation process is typically conducted in conventional resistance furnaces. An interesting alternative to this solution is the use of microwave heating. It helps to obtain materials with better structural properties due to the rapid, uniform, and volumetric heating of the precursor [5,6].

In addition to the structural advantages mentioned above, chemically activated carbons are also characterized by the presence of many functional groups on their surface, which increase the number of possible ways/types of interactions between the adsorbent and the adsorbate, thereby improving the adsorption properties of these materials [7,8]. Thanks to this, activated carbons are commonly used for the removal of a wide range of waterborne contaminants both organic and inorganic, including dyes, pharmaceuticals, pesticides, surfactants, heavy metal ions, and even macromolecular substances such as polymers [9,10,11,12,13,14,15].

The production of activated carbons from plant-based materials such as sawdust, bark and cones of trees [16], nut shells [17], fruit peels [18], or residues from medicinal herb processing (as presented in this study) contributes to the reduction in the amount of agricultural waste requiring landfill storage and to the decrease in greenhouse gas emissions caused by biomass decomposition [19]. Materials obtained from such precursors are commonly referred to as activated biocarbons.

Common sage (*Salvia officinalis* L.) is grown on larger plantations as a herbal plant from which leaves are obtained for food, cosmetic, and medicinal purposes. Unused stems are a waste material that can be used to produce activated biocarbons. The production of this plant in Poland is estimated at about 1500 tons per year. Hence, the amount of residue remaining after its processing is significant.

This paper describes the procedure for producing a series of activated biocarbons from *Salvia officinalis* L. processing residue. Carbonaceous adsorbents were prepared through chemical activation using H_3_PO_4_ and K_2_CO_3_, combined with the application of two heating methods: conventional and microwave-assisted. The resulting materials were comprehensively characterized in terms of their physicochemical and electrokinetic properties, followed by an evaluation of their adsorption capacity toward three selected water pollutants: iodine, methylene blue (a heterocyclic aromatic dye), and Triton X-100 (a nonionic surfactant).

Methylene blue is one of the most popular cationic dyes that is environmentally persistent, toxic, carcinogenic, and mutagenic. It is commonly used for dyeing fabrics in the clothing, textile, paper, and leather industries. Due to the magnitude of industrial usage, a large volume of methylene-blue-containing wastewater is produced, posing a real threat to groundwater and surface water. There are many reports in the literature on the removal of this dye, so the relevant data can be easily compared. In turn, it should be emphasized that there are no reports in the literature concerning Triton X-100 removal with the use of biochars and activated biocarbons. The presented results indicated that the sorption capacities towards this nonionic surfactant are the greatest among all the carbonaceous materials examined in our group. This is particularly important in the context of the high toxicity of Triton X-100 to living organisms, and the effects of its impact on the aquatic environment are long-term. For this reason, regeneration and reusability studies (adsorption cycles) were performed for this hazardous organic substance.

## 2. Results and Discussion

### 2.1. Effect of the Chemical Activation Procedure on Mass Yield and Ash Content

**Figure 1:**
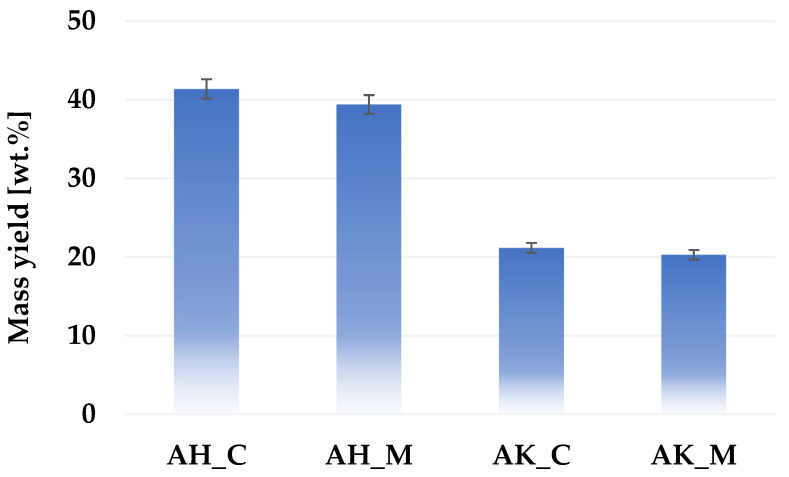
Mass yield of activated biocarbons obtained from sage stems under different thermochemical treatment conditions.

Figure 1 presents a comparison of the mass yield of activated biocarbons produced via different chemical activation variants applied to sage stems. The results indicate that activation with orthophosphoric acid leads to significantly higher product yields compared to activation with potassium carbonate. A slight advantage in mass yield was also observed for the conventional heating procedure.

**Figure 2:**
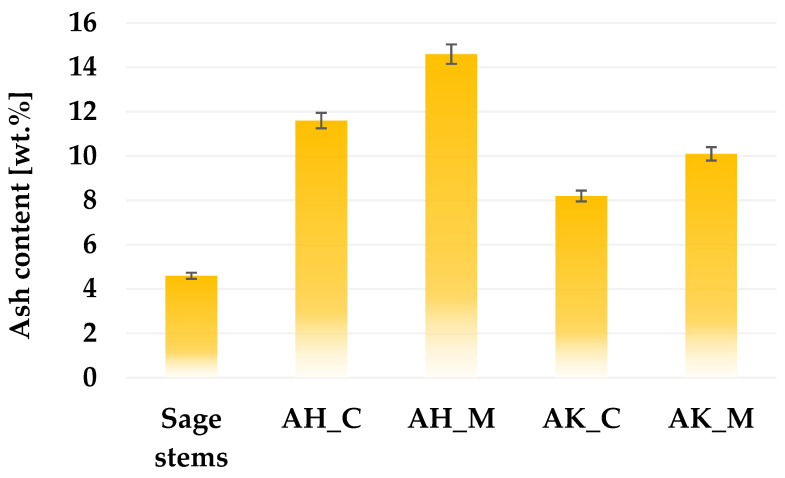
The ash content in activated biocarbons derived from sage stems using different thermochemical treatment methods.

According to the data presented in Figure 2, the content of mineral admixtures (ash) in the structure of the prepared activated biocarbons is largely determined by the activation and post-treatment procedure. The application of each thermochemical treatment variant to sage stems led to an increase in ash content; however, this increase was considerably more pronounced for samples activated with orthophosphoric acid. The observed differences in ash content between acid- and alkaline-activated biocarbons can be attributed to both the chemical nature of the activating agents and the subsequent washing procedure. This is likely due to the incorporation or retention of phosphorus-containing mineral residues (e.g., phosphate salts) that are not easily removed during rinsing, especially if the rinsing is mild. This assumption is supported by the XRF analysis results (Table 1), which revealed a significantly higher phosphorus content in acid-activated samples. In contrast, the alkaline-activating agent may react with silicates or other mineral phases and form alkali-metal compounds that remain in the structure after heat treatment, which may increase the ash content. However, as mentioned earlier, a two-step washing procedure was applied for the K_2_CO_3_-treated samples. The use of boiling hydrochloric acid in the first stage of this procedure contributes to a significant reduction in the content of mineral matter in the final product’s structure (which was also confirmed by the XRF study described later). Although it increases the overall production cost, this step is necessary because skipping it will lead to blocking a significant part of the pores created during activation.

### 2.2. Elemental Composition and Surface Morphology of the Activated Biocarbons Obtained from Sage Stems

According to the XRF analysis results (Table 1), raw sage stems contain a number of inorganic elements with a predominance of potassium (8.2 mg/g) and calcium (6.9 mg/g). The presence of these elements indicates the plant’s natural ability to accumulate them and may also be related to the presence of carbonates and mineral salts from the soil. The relatively high silicon content (2.1 mg/g) may be related to the presence of soil-derived silicates or mineral contaminants. Small amounts of phosphorus (0.4 mg/g), sulfur (0.3 mg/g), and chlorine (0.7 mg/g) were also detected in the samples, which may originate from fertilizers or the plant’s natural metabolic processes. The content of metals such as iron (0.5 mg/g), copper (0.1 mg/g), and manganese (0.1 mg/g) was low, while elements such as nickel and chromium were not detected (<0.1 mg/g), confirming the low contamination of the raw material with transition metals. The elemental composition of the sage stems suggests that this material is a clean and ecological plant material, is suitable for further activation processes, and has minimal risk of undesirable inorganic admixtures.

After activation, a substantial decrease in most inorganic components was observed, particularly for potassium and calcium. However, a significant increase in phosphorus content was recorded in the samples activated with phosphoric acid (AH_C and AH_M), reaching 15.2 mg/g and 8.2 mg/g, respectively. This suggests partial retention of the activating agent, especially in conventionally heated samples, despite the post-activation washing procedure. The iron content also increased notably in sample AH_C (4.8 mg/g), along with detectable levels of chromium (1.1 mg/g) and nickel (2.5 mg/g). These elements probably originated from impurities introduced during thermal treatment, possibly due to the partial degradation or leaching of the nickel steel boat under high-temperature conditions in the presence of orthophosphoric acid. In contrast, no such metal contamination was observed in the corresponding microwave-heated sample (AH_M), which was processed in quartz crucibles, indicating that the choice of reactor material plays a critical role in minimizing contamination. Samples activated with potassium carbonate (AK_C and AK_M) showed significantly lower phosphorus concentrations (0.1–0.3 mg/g), which indicates a clear effect of the activating agent on the residual elemental composition of the biocarbons. However, the influence of the heating method seems to be less significant.

The elemental composition of the activated biocarbon samples (Table 2) indicates that the final chemical structure is influenced by both the activating agent and the heating method. Samples activated with phosphoric acid (AH_C and AH_M) showed greater variability in composition, with AH_C exhibiting the highest carbon content (85.3 wt.%) and lowest oxygen content (11.1 wt.%), while AH_M had significantly less carbon (71.9 wt.%) and much more oxygen (24.2 wt.%). In contrast, the potassium carbonate-activated samples (AK_C and AK_M) showed more consistent carbon content (79.8 and 78.2 wt.%, respectively) and moderately increased oxygen levels compared to AH_C. In general, conventional heating tends to produce biocarbons with higher carbon and lower oxygen contribution, regardless of the activating agent. Nitrogen and hydrogen levels remained low and relatively stable across all samples.

**Figure 3:**
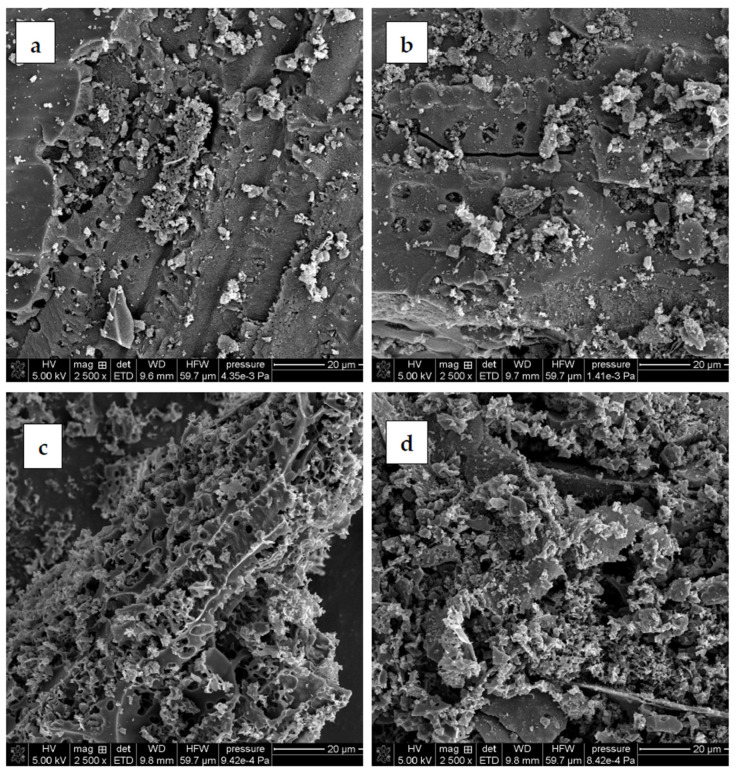
SEM images of activated biocarbons: (**a**) AH_C, (**b**) AH_M, (**c**) AK_C, and (**d**) AK_M.

The surface morphology of carbonaceous materials derived from sage stems is presented in Figure 3. An analysis of the SEM images shows that the activation products differ significantly in terms of morphology. Noticeable differences are observed in pore characteristics, including size, shape, and distribution. The effect of the activating agent is clearly visible in the surface morphology, while the effect of the heating variant is not distinctly marked. At first glance, porosity appears to be better developed in the samples activated with potassium carbonate. These materials exhibit a more complex network of smaller pores. In contrast, samples treated with phosphoric acid display a substantially higher presence of light-colored and small particles, most probably corresponding to mineral impurities. SEM analysis, therefore, confirms the effectiveness of the two-step washing procedure—in particular, the initial HCl treatment—in removing ash from the porous structure.

### 2.3. Textural Parameters of the Activated Biocarbons Derived from Sage Stems

From an adsorption point of view, one of the most important parameters of activated biocarbons is the type of porous structure developed during activation. Data summarized in Table 3 demonstrate that both the type of activating agent and the heating method applied during the activation stage have a significant influence on the development of the porous structure of carbonaceous materials derived from sage stems.

Both samples activated with orthophosphoric acid exhibit significantly higher specific surface areas, particularly the AH_M sample, which was heated by microwaves and reached a surface area of 1151 m^2^/g. In contrast, the corresponding carbon material obtained by activation with K_2_CO_3_ showed a value more than two times lower of 471 m^2^/g. However, the poorest textural parameters were recorded for the AK_C sample, obtained by conventional heating.

Such unfavorable textural parameters of samples activated with potassium carbonate suggest that the conditions of the activation procedure were suboptimal. It would probably be necessary to increase the activation temperature, extend the thermal treatment time, or even increase the activator–precursor weight ratio to 3:1 or 4:1. However, such modifications would inevitably lead to a significant increase in the production cost.

The obtained data also indicate that the activation of sage stems with potassium carbonate favors the development of micropores in the structure of resulting activated biocarbons, whereas treatment with H_3_PO_4_ predominantly leads to the formation of mesopores. Furthermore, the application of microwave heating increases the proportion of micropores in carbonaceous materials produced using both activating agents. The results also indicate that the synergistic effect of H_3_PO_4_ activation and microwave-assisted heating provides the most favorable conditions for producing high-quality activated biocarbons from sage stems.

### 2.4. Acid Base Properties of the Activated Biocarbons Derived from Sage Stems

According to the data collected in Table 4, the stems of sage used in this study are characterized by a high content of both acidic and basic functional groups, with a slight predominance of the acidic ones. The carbonaceous materials obtained via thermochemical conversion of the precursor differ significantly in terms of the chemical character. Both samples activated with H_3_PO_4_ show a strongly acidic nature, with pH values ranging from 2.32 to 2.50, and their surfaces are exclusively covered with acidic functional groups. As can also be seen, the AH_M sample (produced using microwave-assisted heating) contains a significantly higher concentration of these functional groups. In contrast, the analogous biocarbons activated by potassium carbonate exhibit a slightly acidic nature, with pH values changing in the range of 5.45–5.73. Moreover, both acidic and basic functional groups are present on their surface; however, acidic groups predominate.

### 2.5. Surface and Electrokinetic Properties of the Activated Biocarbons Derived from Sage Stems

**Figure 4:**
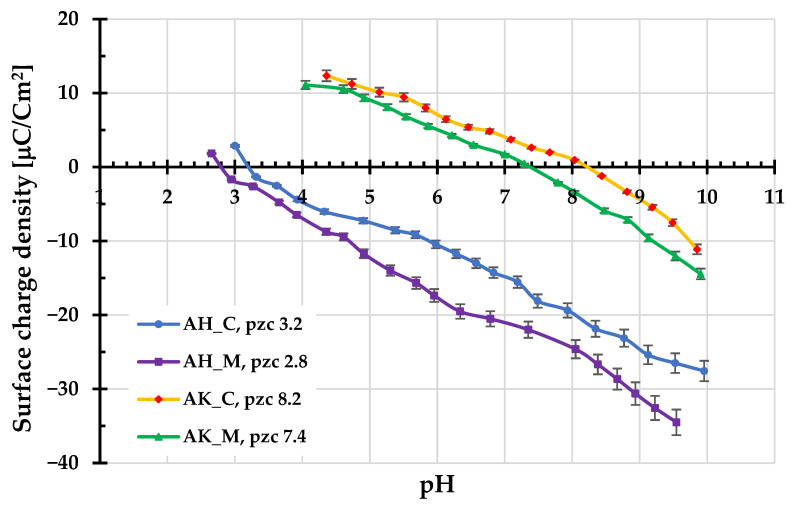
Surface charge density of activated biocarbons as a function of solution pH.

**Figure 5:**
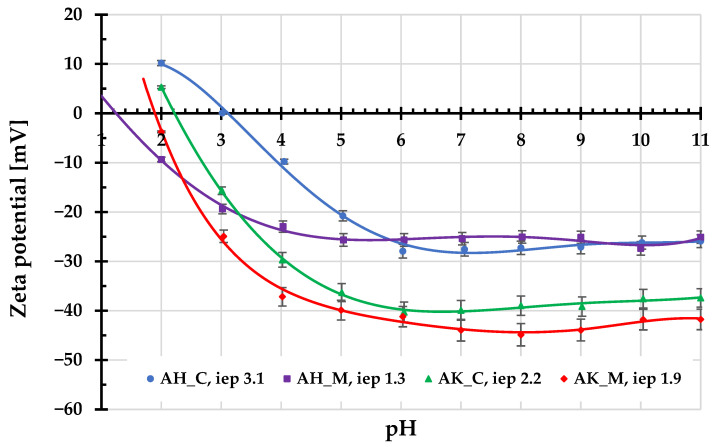
Zeta potential of activated biocarbons particles as a function of solution pH.

Figure 4 and Figure 5 present the results of potentiometric titrations and electrophoretic mobility measurements for the aqueous suspensions of activated biocarbons. As shown in Figure 3, materials obtained using H_3_PO_4_ as the activating agent are characterized by the considerably lower values of the surface charge density (σ_0_) parameter in the whole range of studied pH compared to those activated with K_2_CO_3_. The corresponding pH_pzc_ values for AH_C and AH_M samples are located in the acidic pH range, at 3.2 and 2.8, respectively. In contrast, the pH_pzc_ values for the AK_C and AK_M materials are located at 8.2 and 7.4, respectively. This means that, in the case of carbonaceous adsorbents prepared via chemical activation with ortophosphoric acid, negatively charged surface groups predominate over a wide pH range (approximately from a pH of about 3 to 10). A similar situation occurs in the case of porous materials activated by potassium carbonate, but only within a narrower pH range of 7–8. 

The surface charge density provides information about both the sign and amount of the charges accumulated in the surface Stern layer within the edl. This parameter directly affects the adsorption properties of a given solid regarding the electrostatic interactions. In turn, the zeta potential value reflects the sign and amount of the charges present in the by-surface layer of edl (more precisely in the slipping plane area), the position of which determines the beginning of the diffusion layer. The specific value of zeta potential is of great importance for the stability of solid particles suspensions, as it assures the effective repulsion between them, providing electrostatic stabilization. Consequently, the ionic compositions of the surface and diffusion layers may differ significantly, as evidenced by the course of the zeta potential curves presented in Figure 5.

For all examined activated biocarbons, the zeta potential assumes negative values (except for the first two starting values). The isoelectric points are in the range of 1.3–3.1, indicating that they occur at very acidic pHs. The change in ionic composition of the edl diffusion part is mainly the result of the overlapping of these edl parts within the porous structure of the adsorbents [20]. 

The mean aggregate sizes determined for AH_C, AH_M, AK_C, and AK_M samples are 6030, 557, 630, and 442 nm. They were measured at the natural pHs of suspensions (approximately 4.0–5.5) immediately after their preparation. Only in the case of the AH_C sample was a significantly larger size (about ten times bigger than for other activated biocarbons) of aggregates observed. This can be associated with the relatively small absolute value of the zeta potential at pH 4, which does not guarantee the effective electrostatic stabilization of the solid particles, as a result of which they aggregate by coagulation. For other systems, the absolute value of zeta potential exceeds 20 mV, which significantly improves the dispersion of particles in the aqueous suspension.

### 2.6. Adsorption Properties of the Activated Biocarbons Derived from Sage Stems

**Figure 6:**
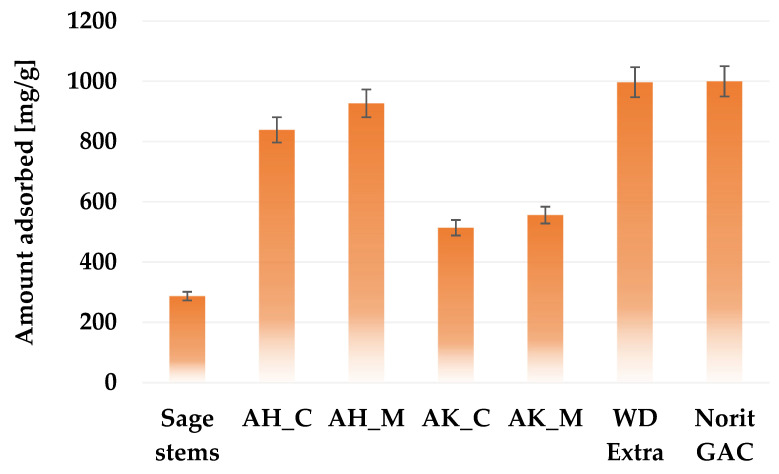
Iodine adsorption capacity of activated biocarbons derived from sage stems compared with selected commercial activated carbon products.

In order to evaluate the sorption capacity of the obtained activated biocarbons and assess their suitability for removing small-molecule impurities from the aqueous solutions, the iodine number was determined. According to the data presented in Figure 6, the precursor used for the study is able to adsorb almost 290 mg of iodine, which means that it can be used as a bioadsorbent. However, it should be emphasized that each of the applied chemical activation variants significantly improves the adsorption capacity. Biocarbons activated with orthophosphoric acid show significantly better sorption properties for iodine than samples treated with potassium carbonate, which is of course a consequence of their much better developed porous structure (Table 3). A clear relationship can be also observed between the heating method applied during activation and the iodine adsorption. Samples heated in a microwave furnace adsorbed several dozen milligrams more iodine than those heated in a conventional way. It can also be seen that materials with a higher content of acidic surface functional groups (Table 4) exhibit greater efficiency in removing iodine from aqueous solutions. The highest iodine sorption capacity (927 mg/g) was noted for the AH_M sample. This result is comparable to the adsorption performance of commercially available activated carbons derived from coal, such as Chem WD-ekstra (997 mg/g) and Norit GAC 1240 W (950 mg/g).

**Figure 7:**
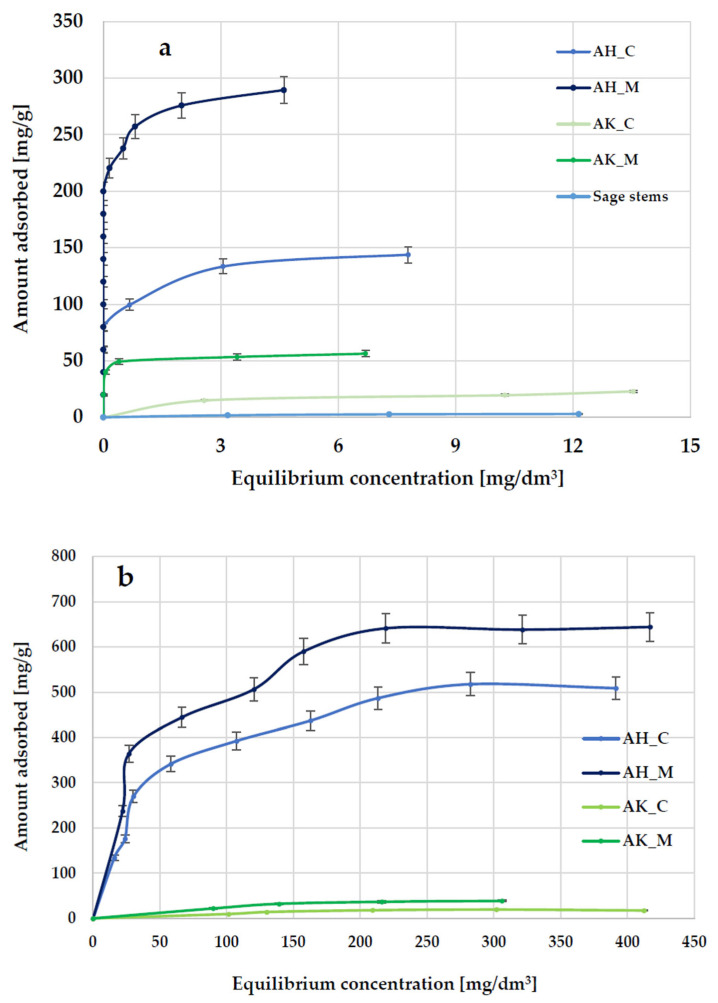
The equilibrium isotherms of methylene blue (**a**) and Triton X-100 (**b**) adsorption from aqueous solution.

Another method for evaluating the sorption properties of activated biocarbons derived from sage stems was the assessment of their efficiency in removing organic contaminants from aqueous solutions using methylene blue as a model synthetic thiazine dye and Triton X-100 as a model surfactant. The results demonstrated that raw waste biomass, in the form of ground sage stems, exhibits poor adsorption performance toward methylene blue and is, therefore, unsuitable for use as an effective adsorbent. However, chemical activation—especially via orthophosphoric acid treatment—significantly improves its adsorption properties and is considered economically viable. The data presented in Figure 7 and Table 5 indicate that, similarly to iodine adsorption, significantly higher sorption capacities for methylene blue and Triton X-100 are exhibited by materials activated with orthophosphoric acid. The most effective adsorbent was the AH_M sample, which adsorbed up to 289.6 mg of the dye and 644.3 mg of the surfactant per gram of adsorbent. Furthermore, the influence of the heating method on the adsorption performance of the obtained biocarbons was, again, visible. Carbonaceous materials heated in a microwave-assisted manner (for both activating agents) showed significantly higher adsorption capacity compared to both conventionally heated samples and the raw sage stems. 

**Figure 8:**
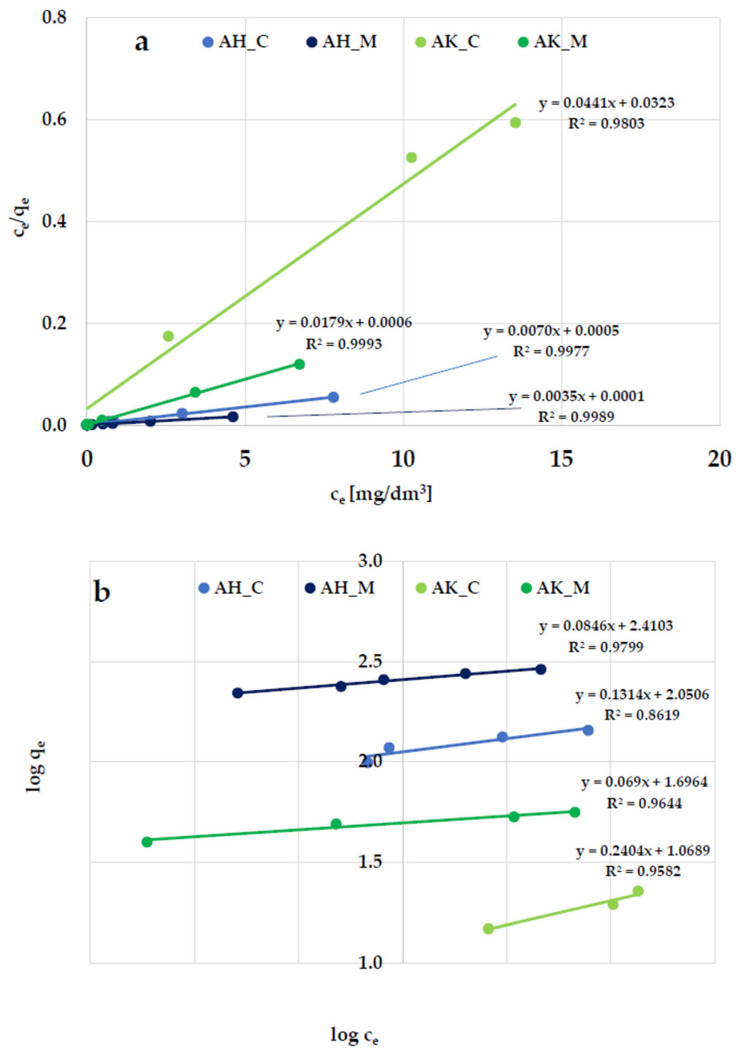
Linear fitting of methylene blue adsorption isotherms to the (**a**) Langmuir and (**b**) Freundlich models.

**Figure 9:**
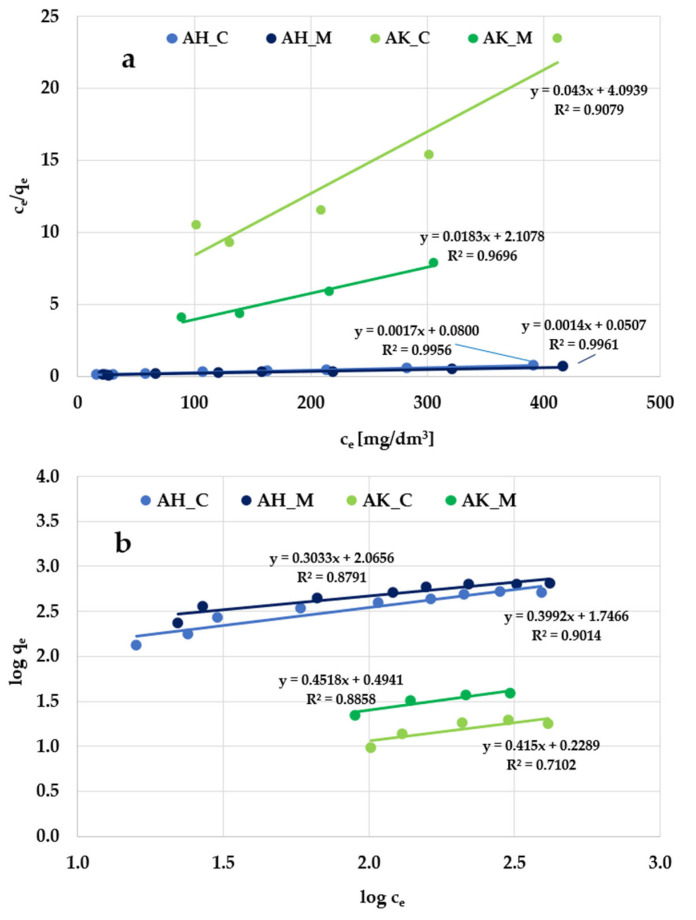
Linear fitting of Triton X-100 adsorption isotherms to the (**a**) Langmuir and (**b**) Freundlich models.

According to the data presented in Figure 8 and Figure 9, and Table 5, the Langmuir isotherm fits the experimental data more accurately than the Freundlich model, so it can be assumed that methylene blue and Triton X-100 adsorption most likely take place in the form of a monolayer coating of the activated biocarbon surface with dye molecules. This is supported by both the higher (close to 1) values of the determination coefficient (R^2^) and the comparable values of experimentally determined (q_exp_) and calculated (q_m_) sorption capacities. However, the relatively high R^2^ values observed in the case of the Freundlich isotherm model (ranging from 0.936 to 0.980) obtained for methylene blue adsorption suggest that a more complex adsorption mechanism—potentially involving multilayer adsorption—is also possible.

**Figure 10:**
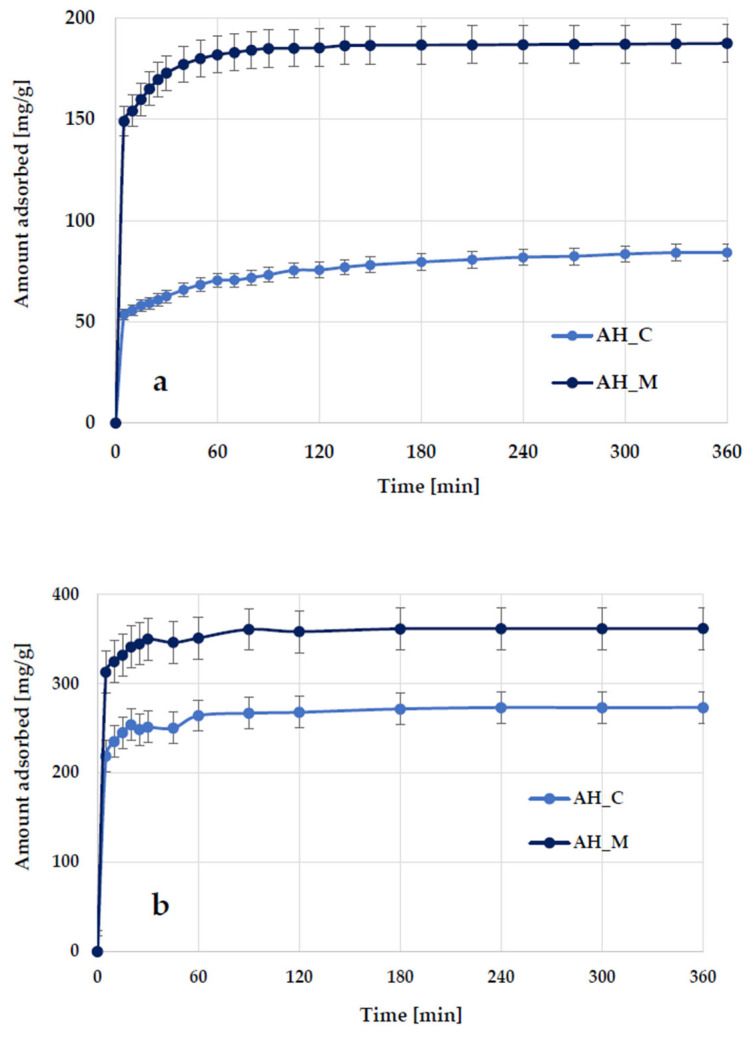
Effect of phase contact time on methylene blue (**a**) and Triton X-100 (**b**) adsorption on the H_3_PO_4_-activated biocarbons derived from sage stems.

The data illustrating the effect of contact time on methylene blue and Triton X-100 adsorption on activated biocarbons derived from sage stems (Figure 10) indicate that the adsorption kinetics consist of two main stages: an initial rapid phase, during which adsorption occurs very quickly and significantly contributes to the equilibrium uptake of methylene blue/Triton X-100, followed by a slower second step, which contributes less to the total dye/surfactant adsorption. The first stage most likely corresponds to the instantaneous adsorption of methylene blue/Triton X-100 or the rapid coverage of the external surface of the activated biocarbon by dye/surfactant molecules, while the second stage involves a gradual adsorption process leading to the equilibrium state.

**Figure 11:**
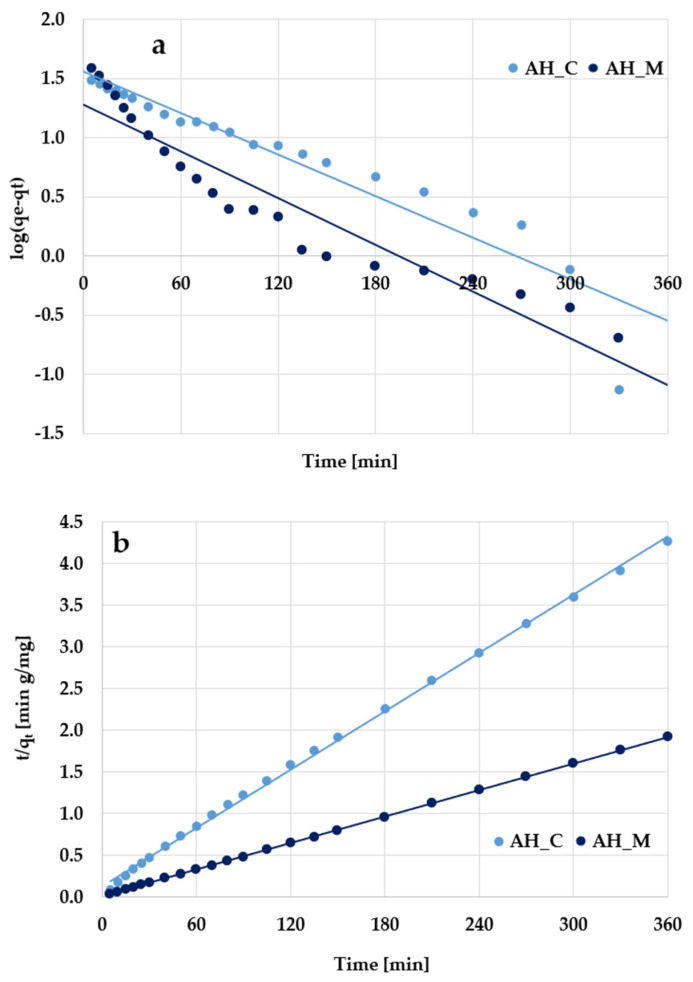
(**a**) Pseudo-first-order and (**b**) pseudo-second-order kinetic plots of methylene blue adsorption on the H_3_PO_4_-activated biocarbons derived from sage stems.

**Figure 12:**
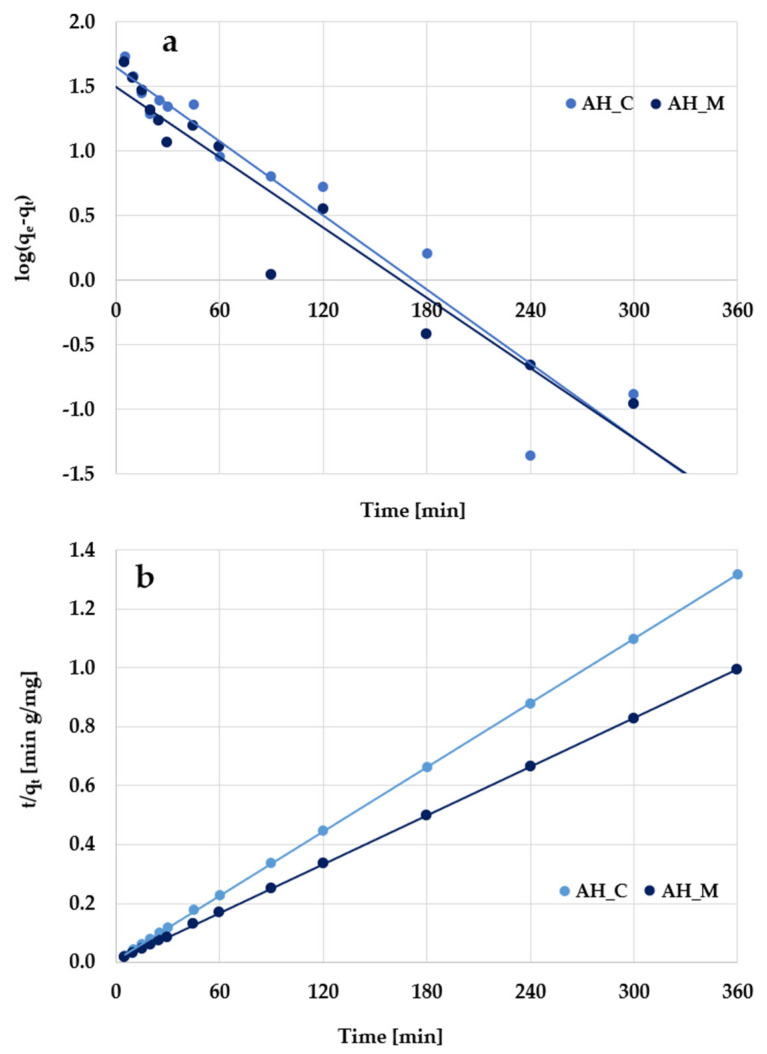
(**a**) Pseudo-first-order and (**b**) pseudo-second-order kinetic plots of Triton X-100 adsorption on the H_3_PO_4_-activated biocarbons derived from sage stems.

According to the kinetic plots presented in Figure 11 and Figure 12 as well as data collected in Table 6, the experimental results are better described by the pseudo-second-order kinetic model, as evidenced by the high determination coefficients (R^2^) ranging from 0.998 to 0.999. The pseudo-second-order kinetics is further supported by the close agreement between the calculated adsorption capacities (q_cal_) and the experimentally determined values (q_exp_). Based on these findings, it can be supposed that methylene blue/Triton X-100 adsorption primarily involves chemical interactions with the surface of activated biocarbons derived from sage stems.

**Figure 13:**
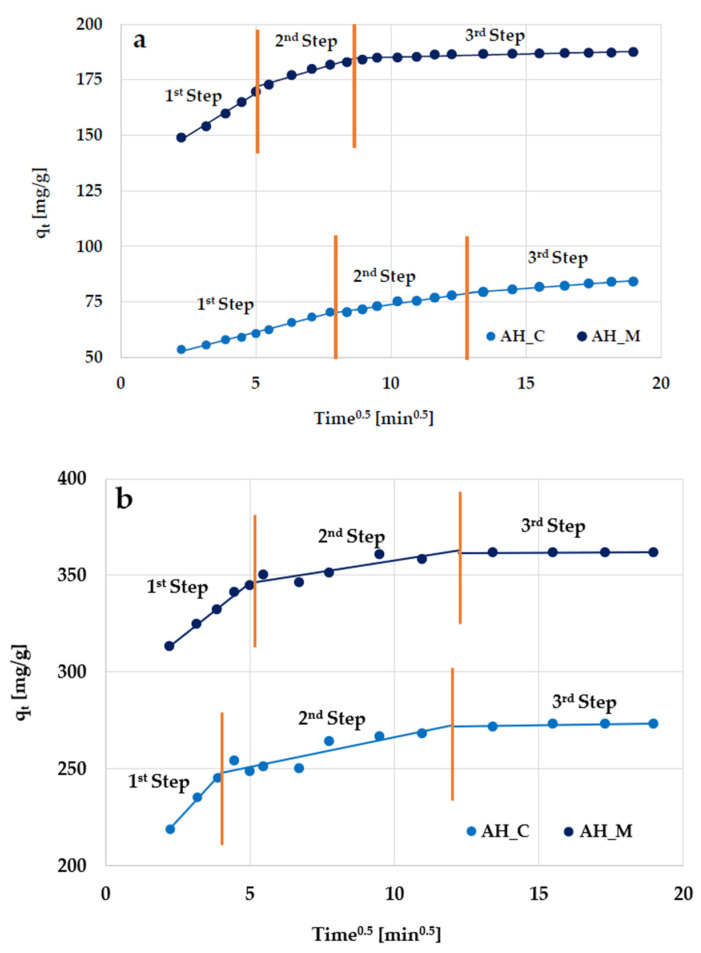
Intraparticle diffusion model for (**a**) methylene blue and (**b**) Triton X-100 adsorption on the H_3_PO_4_-activated biocarbons derived from sage stems.

In order to explain the mechanisms of the transport and diffusion of adsorbate molecules during their adsorption on activated biocarbons, the intraparticle diffusion model was used [21,22]. The dependencies of q_t_ versus t^0.5^ for the adsorption of organic substances are presented in Figure 13, and the corresponding data are summarized in Table 7. Linearized forms of the model were fitted to describe each adsorption phase. As can be seen, the adsorption process for the examined substances includes three separate stages.

In the first stage, rapid adsorption occurs (within the first few minutes of contact). This is due to the high availability of active sites on the activated biocarbon’s surface and the high concentration gradient between the molecules of organic substances in the solution and on the adsorbent surface. In this phase, surface adsorption dominates, in which the molecules quickly bind to the available external surface sites on the carbonaceous materials. Internal diffusion limitations do not occur, allowing for considerable adsorption in a short time. In the second stage, adsorption proceeds more slowly because the surface sites have been occupied, limiting the process via intramolecular diffusion into the deeper pores. The penetration of organic molecules into the internal structure increases the resistance to diffusion, limiting the adsorption rate. Finally, in the third stage, the maximum adsorption is achieved for all examined systems. 

The parameters of the intraparticle diffusion model (Table 7) indicate that velocity constants decrease according to k_i1_ > k_i2_ > k_i3_, which confirms that the rate of organic molecule removal is the highest in the first stage of adsorption (especially in the case of Triton X-100).

**Figure 14:**
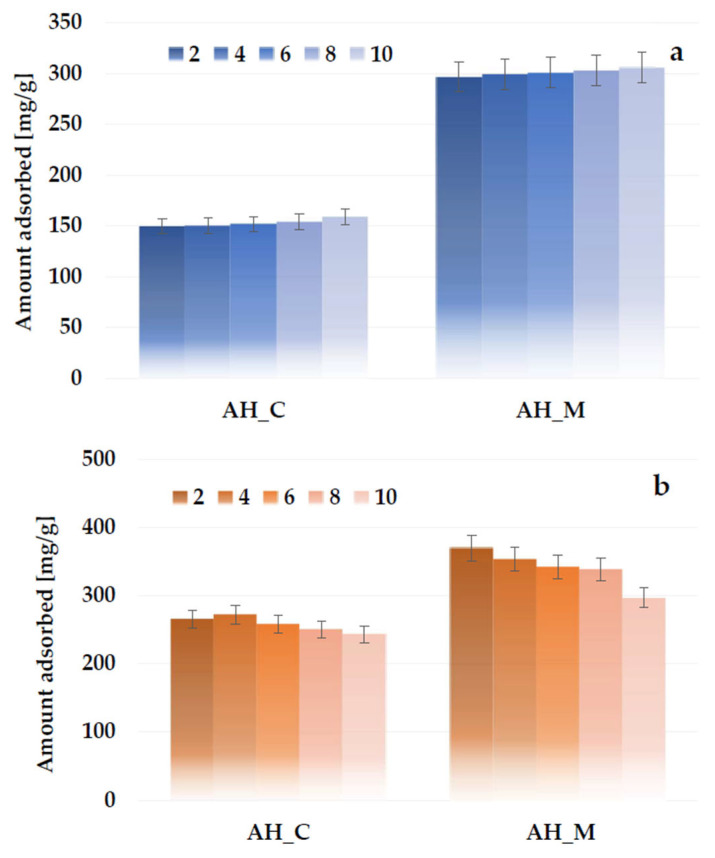
Effect of solution pH on methylene blue (**a**) and Triton X-100 (**b**) adsorption on the H_3_PO_4_-activated biocarbons derived from sage stems.

The analysis of the data presented in Figure 14a indicates that, for both activated biocarbons activated with orthophosphoric acid, the highest adsorption capacity of methylene blue is achieved at pH 10. This may be attributed to the fact that, at higher pH values, the deprotonation of surface functional groups leads to a significant decrease in H⁺ ion concentration, resulting in a negative charge on the adsorbent surface. This enables the electrostatic attraction between the cationic methylene blue molecules and the negatively charged biocarbon surface, which positively affects the adsorption efficiency. In turn, the least favorable results for the AH_C and AH_M samples were obtained at pH 2, with a sorption capacity that was lower by 9.4 mg/g and 8.9 mg/g, respectively. At lower pH values, functional groups present on the adsorbent surface become protonated, so the excess of H^+^ ions can compete with dye molecules for the active sites and, as a consequence, limit their adsorption. However, it should be noted that the effect of the solution pH on the sorption capacity of the biocarbons derived from sage stems is considerably less significant than the impact of the activation method or the heating variant.

The opposite pH dependence was observed for Triton X-100 adsorption (Figure 14b): the amount of adsorbed surfactant decreases slightly with the pH increase. Despite the nonionic nature of the surfactant, its molecule can be divided into two parts with different hydrophilic-hydrophobic properties. There are hydrophobic substituents, including a phenolic ring (head), and a hydrophilic polyoxyethylene group (tail). In contrast to the cationic dye, the deprotonation of the activated biocarbon surface groups with increasing solution pH has an adverse effect on the formation of, e.g., hydrogen bonds with the hydrophilic part of the surfactant.

The adsorption capacities of other activated biocarbons towards methylene blue and Triton X-100 are presented and compared with those obtained for AH_C and AH_M samples (Table 8). The analysis of the above data indicates that the sorption capacities of AH_C- and AH_M-activated biocarbons in relation to the cationic dye remain at an intermediate level in relation to the carbonaceous materials described in scientific papers [21,22,23,24,25,26,27]. In the case of Triton X-100, there are no data in the literature on its removal using biocarbons and activated biocarbons apart from studies conducted in our research group [28,29,30]. Both carbonaceous materials obtained from sage stems via chemical activation with H_3_PO_4_ show significantly higher sorption capacities than those obtained by us from other precursors.

**Figure 15:**
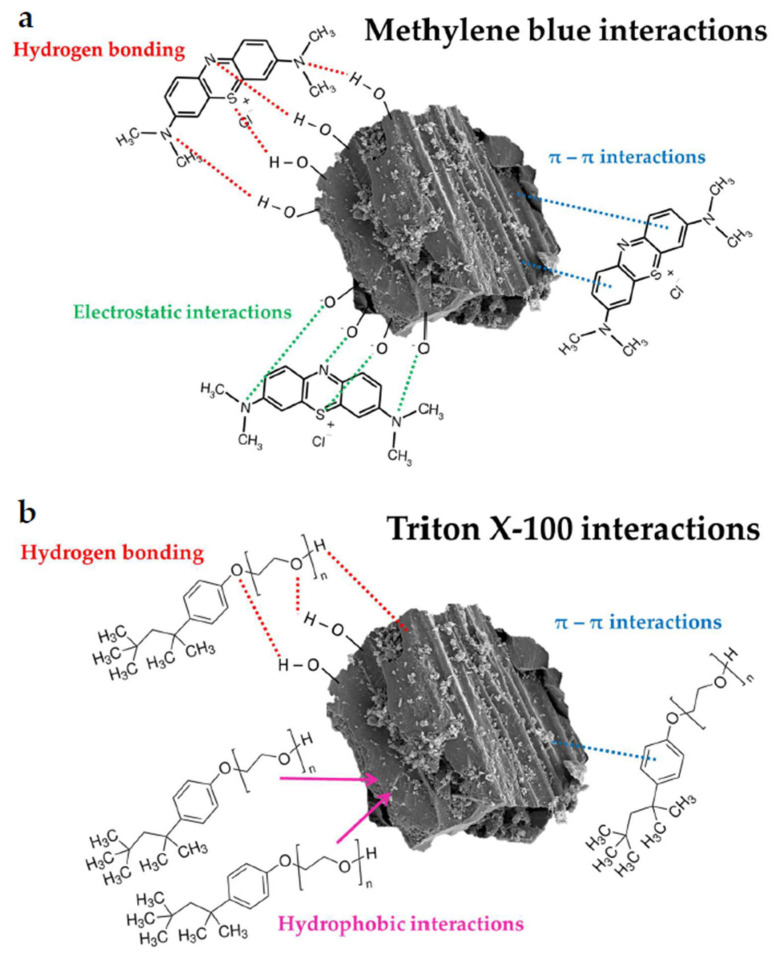
The schematic representation of possible interactions in the examined systems containing methylene blue (**a**) and Triton X-100 (**b**).

The interactions shown in Figure 15 are most likely responsible for the adsorption of methylene blue and Triton X-100 on the surface of the obtained activated biocarbons.

### 2.7. Thermodynamics of Organic Molecule Adsorption on the Activated Biocarbons’ Surface

**Figure 16:**
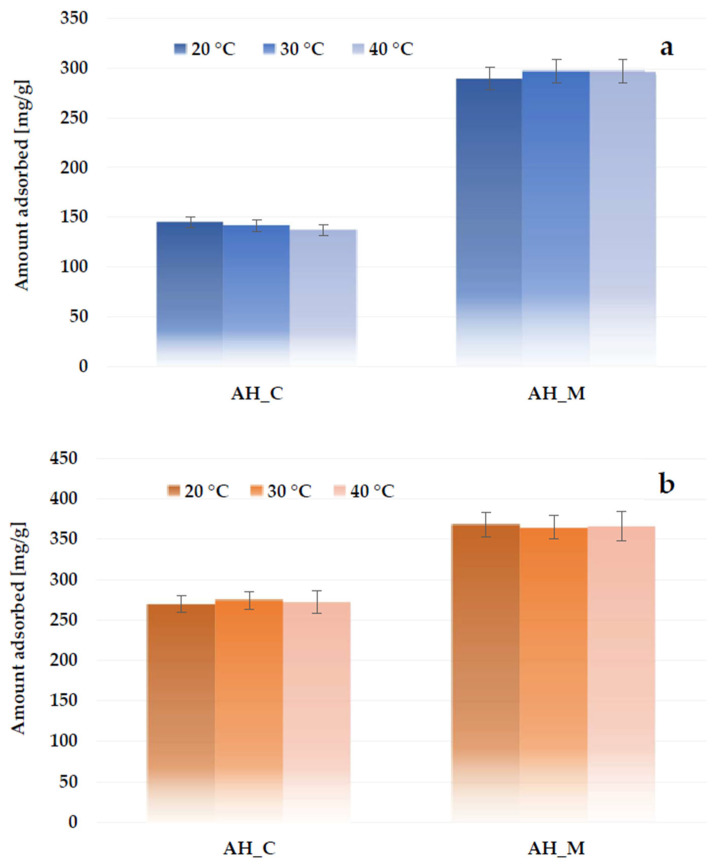
Effect of temperature on methylene blue (**a**) and Triton X-100 (**b**) adsorption on the H_3_PO_4_-activated biocarbons derived from sage stems.

The data presented in Figure 16 show that the effect of system temperature on adsorption efficiency is not very significant. The sorption capacity of the AH_C sample decreases slightly (by 7.8 mg/g) as the solution temperature increases from 20 °C to 40 °C. In contrast, the sorption capacity of the material activated in a microwave furnace increases by 7.5 mg/g with rising temperature. In the case of nonionic surfactants, these differences are even smaller.

**Figure 17:**
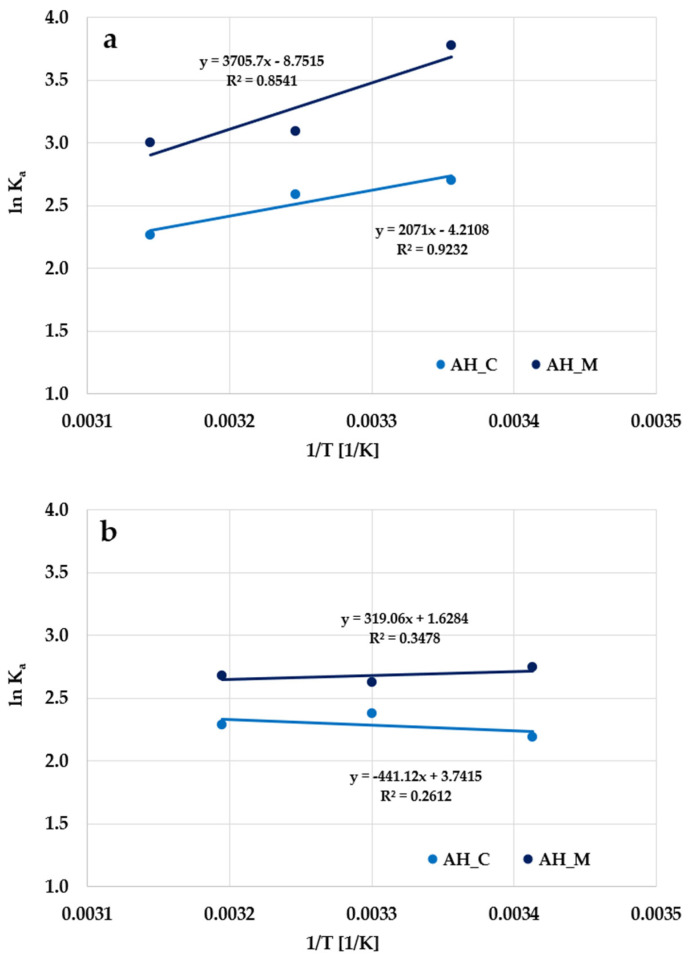
Plot of the adsorption equilibrium constant (lnK_a_) vs. temperature (1/T) for the adsorption of methylene blue (**a**) and Triton X-100 (**b**) on the H_3_PO_4_-activated biocarbons derived from sage stems.

Figure 17 shows the linear dependencies of the adsorption equilibrium constant (lnK_a_) vs. temperature (1/T). The entropy and enthalpy were evaluated from their intercept and gradient, assuming that they are independent of temperature in the studied range (Table 9). The negative values of ∆G^0^ proved that the adsorption process of organic molecules on the activated biocarbons’ surface is spontaneous [31].

The negative values of ∆S^0^ observed for methylene blue adsorption indicate a decrease in randomness at the solid-liquid interface, suggesting a more ordered system during the uptake of the basic dye. It may promote the desorption of dye molecules and their transfer from the solid phase back into the solution. As a result, fewer adsorbate molecules are permanently bound to the surface, leading to the reduced sorption efficiency of the material. In contrast, the positive ∆S^0^ values obtained for Triton X-100 indicate a high affinity of the adsorbent surface for surfactant molecules and may be attributed to the increased interfacial disorder caused by the structural reorganization of surfactant molecules during adsorption. During adsorption, water molecules are displaced by Triton X-100 molecules from the surface of the adsorbent, leading to an overall increase in system entropy.

Adsorption in an activated biocarbon-liquid system involves two simultaneously occurring processes: the desorption of solvent molecules previously adsorbed on the surface of the adsorbent (in this case, water) and the adsorption of adsorbate molecules. In an exothermic process, the total energy required to break the existing bonds (e.g., with water) is lower than the energy released upon the formation of new bonds between the adsorbate and the surface of the adsorbent. As a result, the excess energy is released as heat, leading to negative ∆H^0^ values. This behavior is observed for both materials during methylene blue removal and specifically for the AH_M sample in the case of Triton X-100 adsorption. In an endothermic adsorption process, adsorbate molecules need to displace multiple water molecules from the surface, which requires additional energy. This higher energy demand results in positive enthalpy values (∆H^0^). This type of process occurs during the adsorption of surfactant on the AH_C sample. 

### 2.8. Regeneration and Reusability Studies of Activated Biocarbons in the Systems Containing Triton X-100

**Figure 18:**
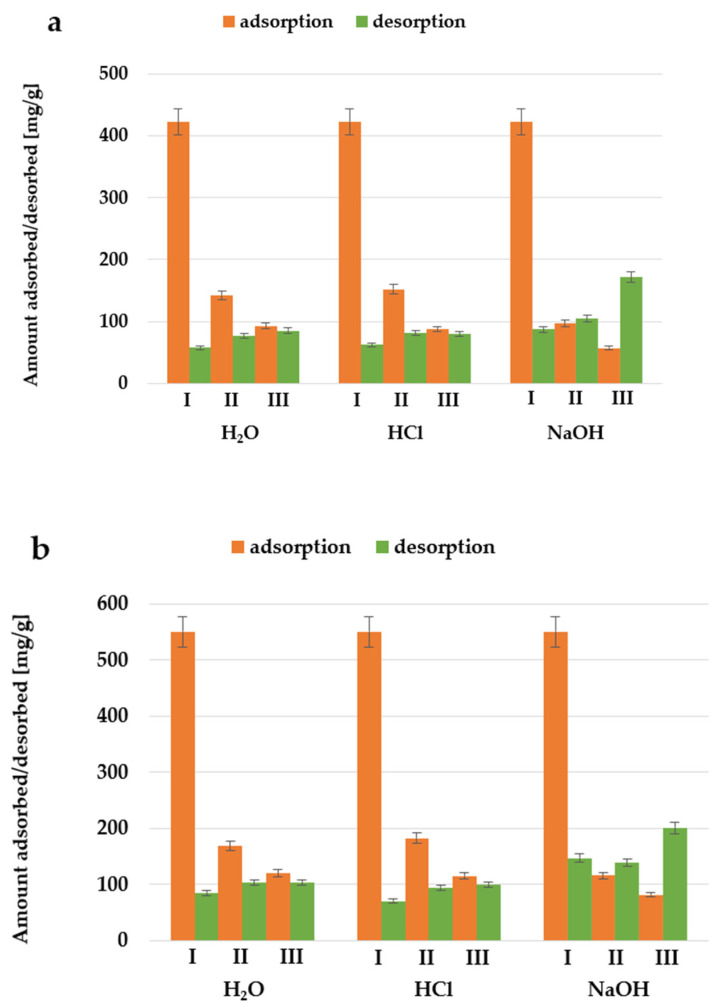
The amounts of Triton X-100 adsorbed and desorbed during three subsequent adsorption–desorption cycles performed for activated biocarbon AH_C (**a**) and AH_M (**b**) using different desorbing reagents.

As shown in Figure 18, regardless of the desorbing agent used, the amount of adsorbed nonionic surfactant significantly decreases with each subsequent cycle. At the same time, an increase in the amount of adsorbate desorbed from the surface of activated biocarbons in subsequent adsorption-desorption cycles is noticeable. Among the desorbing reagents used, the most effective was the NaOH solution, but this variant of desorption is still not satisfactory. It is also worth considering the use of other substances that would interact more strongly with the adsorbent and/or adsorbate, causing the increased desorption of Triton X-100 from the surface of the obtained materials.

## 3. Materials and Methods

### 3.1. Preparation of Activated Biocarbons

The starting material used in the study was sage stems (*Salvia officinalis* L.), cut into pieces approximately 0.5–1.0 cm in length. This material originated from local herbal manufacturers operating in the Lublin region (Poland). Initially, the precursor was divided into four parts. Two of them were impregnated with a 50% H_3_PO_4_ solution (H, EUROCHEM BGD, Tarnów, Poland) at a weight ratio of 2:1 for 24 h at room temperature. The remaining two portions were treated with a K_2_CO_3_ solution (K, EUROCHEM BGD, Tarnów, Poland) using the same weight ratio of the activating agent to the precursor. Then, all samples were dried to constant mass at 110 °C in a laboratory oven (UFP 500 model, Memmert, Büchenbach, Germany). Two of the prepared samples were subsequently subjected to heat treatment in a nitrogen atmosphere (N_2_ 4.0, flow rate 15 L/h; Linde Gaz Polska, Kościan, Poland) using a conventional (C) laboratory furnace equipped with a quartz tube reactor (single-heating-zone model PRW75/LM, Czylok, Jastrzębie-Zdrój, Poland), while the other two were heated in a microwave (M) muffle furnace (Phoenix model, CEM Corporation, Matthews, IL, USA). The thermochemical treatment using microwaves was conducted at a power of 1400 W and a frequency of 2.45 GHz.

The heating procedure was as follows: In the first stage, the samples placed in nickel steel boats (in the case of the conventional furnace) or quartz crucibles (in the case of the microwave furnace) were heated to a temperature of 200 °C at a rate of 5 °C/min and annealed under these conditions for 30 min. In the next stage, the temperature was increased to 550 °C (at the same heating rate) and maintained for another 30 min. After the completion of the heat treatment, the samples were cooled down to room temperature in an inert gas atmosphere. The H_3_PO_4_-activated samples (designated as HA_C and HA_M) were rinsed with 5 dm^3^ hot distilled water in a vacuum filtration funnel (with a glass sintered disc), while the K_2_CO_3_-activated materials (designated as KA_C and KA_M) were first washed with 0.5 dm^3^ of boiling 5% hydrochloric acid (EUROCHEM BGD, Tarnów, Poland) and, subsequently, with hot distilled water until no chloride ions were detected in the filtrate. All samples were then dried at 110 °C to a constant weight. The dried activated biocarbons were weighed to calculate the yield, ground in an agate mortar, and sieved. The fraction with a grain size below 0.125 mm was selected for further studies.

### 3.2. Analytical Procedures

The total content of mineral admixtures (ash) in the structure of the precursor and activated biocarbons was determined by combusting the samples in a microwave muffle furnace (Phoenix, CEM Corporation, Matthews, IL, USA) at 815 °C for 60 min, according to the PN ISO 1171:2002 Standard [32].

The surface morphology of carbonaceous materials was analyzed using a Quanta 250 FEG scanning electron microscope (FEI, Waltham, MA, USA).

The total content of elemental carbon, hydrogen, and nitrogen in the samples was determined using the Vario EL III elemental analyzer (Elementar Analysensysteme GmbH, Langenselbold, Germany). The oxygen content was calculated from the difference. Additionally, the elemental composition of the activated biocarbons was determined using the X-ray fluorescence method (ED-XRF Epsilon 5, PANalytical B.V., Almelo, The Netherlands).

The textural characterization of the activated biocarbons was performed based on the nitrogen adsorption-desorption isotherms measured at −196 °C (ASAP 2020, Micromeritics Norcross, GA, USA). Before the analysis, activated biocarbon samples were degassed under vacuum (at 300 °C) to eliminate any physically adsorbed gases. The total specific surface area of activated biocarbons was calculated using the BET method (within the relative pressure p/p_0_ range of 0.05–0.30). The total pore volume and pore size distribution were determined based on the BJH model, while the micropore volume and area were assessed via the t-plot method.

To characterize the acid base properties of the starting sage stems and the prepared activated biocarbons, the pH value of their aqueous extracts was determined following the procedure detailed in our previous paper [28]. Briefly, 0.5 g of carbonaceous material was added to 0.025 dm^3^ of distilled water and magnetically stirred to reach equilibrium. The pH of the resulting suspension was measured using a CP-401 pH-meter equipped with an EPS-1 combination glass electrode (ELMETRON Zabrze, Poland).

The content of the surface functional groups of basic or acidic nature was determined using the Boehm titration method, described in detail in [33]. Standard solutions of 0.1 mol/dm^3^ NaOH and 0.1 mol/dm^3^ HCl (both from TARCHEM, Tarnowskie Góry, Poland) were used as the titrants. A 1% methyl orange solution (Avantor Performance Materials, Gliwice, Poland) was used as the indicator.

The structure of the electrical double layer (edl) formed around the activated biocarbon particles was characterized by the determination of the following parameters: solid surface charge density (σ_0_), zeta potential (ζ), point of zero charge (pzc), isoelectric point (iep), and mean aggregate size. The first two parameters were obtained via the automated potentiometric titration (Dosimat 765 microburette, Metrohm, Herisau, Switzerland) of activated biocarbons aqueous suspensions using a specialized program “Titr_v3” [34]. In turn, the electrokinetic studies including the determination of the remaining parameters were carried out based on the phenomena of microelectrophoresis and static light scattering using the Zetameter Nano ZS (Malvern Instruments, Cambridge, UK) [35]. Based on the obtained dependencies of the surface charge density and changes in zeta potential as a function of suspension pH, the pH_pzc_ and pH_iep_ points were determined, respectively.

### 3.3. Adsorption Experiments

To determine the sorption properties of the activated biocarbons derived from sage stems, adsorption tests were carried out on three different types of pollutants, i.e., an aqueous iodine solution (representing inorganic pollutants with very small particle sizes of close to 1 nm; Avantor Performance Materials, Gliwice, Poland) and an aqueous solution of methylene blue (a cationic dye representing organic heterocyclic aromatic compounds; Avantor Performance Materials, Gliwice, Poland) as well as an aqueous solution of Triton TM X-100 (representing nonionic surfactants with high molar mass ~ 600 g/mol; Sigma-Aldrich, Saint Louis, MO, USA).

The ability of the activated biocarbons to adsorb iodine from an aqueous solution was determined according to the procedure described in detail in the ASTM D4607-94(2006) Standard [36]. Briefly, 0.2 g of the biocarbon sample was added to 0.02 dm^3^ of 0.1 mol/dm^3^ iodine solution, vigorously shaken, filtered through filter paper, and titrated with 0.1 mol/dm^3^ sodium thiosulphate (Avantor Performance Materials, Gliwice, Poland) in the presence of starch indicator.

The study of sorption capacity towards methylene blue (MB) was carried out according to the following procedure: Equal portions (0.025 g each) of the prepared activated biocarbons were added to 0.05 dm^3^ of MB aqueous solutions (Avantor Performance Materials, Gliwice, Poland) with the initial concentrations ranging from 5 to 150 mg/dm^3^. The suspensions were magnetically stirred at 250 rpm at room temperature overnight to reach equilibrium. Subsequently, the suspensions were centrifuged at 10 000 rpm for 10 min using a Frontier FC5515 micro-centrifuge (OHAUS, Parsippany, NJ, USA) to minimize the presence of carbon particles in the analytes collected for spectroscopic analysis. MB concentration was determined using a Cary 100 Bio double beam UV-Vis spectrophotometer (Varian, Palo Alto, CA, USA) at a wavelength of 665 nm with quantification based on the previously prepared calibration curve. Distilled water was used as the reference.

The adsorption isotherms of the nonionic surfactant on the activated biocarbon’s surface were determined based on changes in the adsorbate concentration before and after the adsorption process using the static method. The examined range of initial Triton X-100 concentrations was 10–800 mg/dm^3^. The mass of activated biocarbons (which was added to the 0.01 dm^3^ of the adsorbate solutions) was chosen individually for each carbonaceous material depending on its specific surface area. Triton TM X-100 concentration was determined at a wavelength of 270 nm [37] using the UV-VIS spectrophotometer Cary 100 Bio (Varian, Palo Alto, CA, USA).

The amounts of organic pollutants adsorbed at equilibrium state (q_exp_, mg/g) were calculated from the following Formula (1):

(1) $$q_{exp}=\frac{∆c\cdot V}{m_{bio}}\;$$

where Δc is the difference between the initial and equilibrium adsorbate concentration [mg/dm^3^], V is the volume of the adsorbate solution [dm^3^], and m_bio_ is the mass of activated biocarbon used during the adsorption test [g].

The experimental adsorption data were modeled using the Langmuir (2) and Freundlich isotherm Equations (3):

(2) $$q_{eq}=\frac{q_{m}K_{L}c_{eq}}{1+K_{L}c_{eq}}$$

(3) $$q_{eq}=K_{F}c_{eq}^{1/n}$$

where q_eq_ is the amount adsorbed at equilibrium [mg/g], q_m_ is the maximal adsorbed amount [mg/g], K_L_ is the Langmuir constant [dm^3^/mg], c_eq_ is the equilibrium concentration of adsorbate [mg/dm^3^], K_F_ is the Freundlich constant [mg/g (mg/dm^3^)^1/n^], and n is the Freundlich parameter.

In the case of the HA_C and HA_M samples activated with orthophosphoric acid (characterized by significantly better adsorption properties), the effects of pH and temperature on the adsorption efficiency of methylene blue and Triton X-100, as well as the adsorption kinetics, were also determined.

The procedure for kinetic studies was similar to that used for the equilibrium tests. The suspensions were magnetically stirred at 250 rpm for 6 h at a temperature of 25 °C and pH 6. During the first 2 h, samples were collected every 15 min and then every 30 min. The obtained results were fitted using the pseudo-first-order and the pseudo-second-order kinetic models, expressed by Equations (4) and (5):

(4) $$\frac{{dq}_{t}}{dt}=k_{1}(q_{eq}-q_{t})$$

(5) $$\frac{{dq}_{t}}{dt}=k_{2}{\left(q_{eq}-q_{t}\right)}^{2}$$

with q_eq_—the amount of dye or surfactant adsorbed in the equilibrium state [mg/g], q_t_—the amount adsorbed after time “t” [mg/g], k_1_—the pseudo-first-order rate constant [1/min], k_2_—the rate constant of the pseudo-second-order adsorption [g/(mg·min)].

Additionally, the adsorption process of methylene blue and Triton X-100 on activated biocarbons obtained using orthophosphoric acid was characterized using the intraparticle diffusion model [38], which is described by Equation (6):

(6) $$q_{t}=k_{i}t^{0.5}$$

where qt—the amount adsorbed [mg/g] after time “*t*” [min], *k_i_*—rate constant [mg/g min^0.5^].

The impact of pH on the efficiency of methylene blue and Triton X-100 adsorption was evaluated in the range of 2–10. Prior to each experiment, the solution pH was adjusted by adding appropriate amounts of 0.1 mol/dm^3^ HCl or NaOH (TARCHEM, Tarnowskie Góry, Poland). The pH was measured using CP-401 pH meter equipped with a combined glass electrode.

The effect of the temperature on the adsorption efficiency of the aforementioned organic compounds from the liquid phase was investigated using a Unimax 1010 shaker equipped with an incubator (Heidolph Instruments GmbH & Co. KG, Schwabach, Germany). The suspensions were shaken for 12 h at temperatures of 20, 30, or 40 °C. The obtained results of the dependence of the adsorption on the temperature were used to determine the thermodynamic parameters [39] using Equations (7)–(9):

(7) $$∆G{}^{\circ}=\;-RT\ln K_{a}$$

(8) ∆G°= ∆H°−T∆S°

(9) $$\ln K_{a}=\;-\frac{∆H{}^{\circ}}{RT}\;+\;\frac{∆S{}^{\circ}}{R}$$

where Δ*G*°—Gibbs energy change [J/mol], *R*—gas constant [8.314 J/mol K], *T*—temperature [K], *K_a_*—adsorption equilibrium constant [dm^3^/g], Δ*H*°—enthalpy change [J/mol], Δ*S*°—entropy change [J/mol K].

For the H_3_PO_4_-activated biocarbons derived from sage stems, three adsorption–desorption cycles were carried out to determine their regenerative properties in terms of removing Triton X-100 from aqueous solutions (surfactant initial concentration was 600 mg/dm^3^). Three different reagents were used for desorption: redistilled water, 0.1 mol/dm^3^ HCl, and 0.1 mol/dm^3^ NaOH.

## 4. Conclusions

It has been demonstrated that herbal waste, such as sage stems, can be effectively utilized as a low-cost and environmentally friendly precursor for the production of efficient adsorbents. This study revealed that the adsorption capacity, textural properties, and chemical characteristics of the biocarbon surface are significantly influenced by the type of activating agent used and the thermal treatment method applied. Orthophosphoric acid proved to be a highly reactive activating agent for waste biomass, enabling the production of materials with a well-developed specific surface area and significant pore volume, even at relatively low temperatures such as 550 °C. This is advantageous from both economic and environmental perspectives. The combination of phosphoric acid activation and microwave treatment led to the formation of a mesoporous structure, which is particularly favorable for the adsorption of larger pollutant molecules, such as organic dyes and surfactants. This synergistic approach enhanced both the structural and sorption properties of the material, confirming its potential for the production of efficient bio-based adsorbents. Remarkably, one of the produced biocarbons (sample AH_M) demonstrated an iodine sorption capacity comparable to that of commercial activated carbons. Since iodine adsorption is a well-known indicator of the material’s ability to capture small-sized contaminants (approximately 2 nm), this result highlights the high versatility of the obtained adsorbent. These promising results suggest that further research aimed at optimizing the production process would be valuable, particularly in the context of investigating the effects of activation time and the precursor-to-activating-agent ratio on the physicochemical properties and adsorption performance.

## Figures and Tables

**Table 1:** XRF chemical composition of activated biocarbons obtained from XRF analysis.

Element	Content [mg/g]				
	Sage Stems	AH_C	AH_M	AK_C	AK_M
Si	2.1	0.9	0.7	1.1	2.2
P	0.4	15.2	8.2	0.1	0.3
S	0.3	0.1	0.1	0.1	0.1
Cl	0.7	0.1	0.0	0.3	1.0
K	8.2	0.0	0.1	0.2	0.4
Ca	6.9	2.0	1.9	0.3	0.9
Ti	0.2	0.0	0.1	0.1	0.1
Cr	0.0	1.1	0.0	0.2	0.0
Mn	0.1	0.1	0.0	0.0	0.0
Fe	0.5	4.8	0.3	0.4	0.3
Ni	0.0	2.5	0.0	0.1	0.0
Cu	0.1	0.5	0.1	0.1	0.6

**Table 2:** The elemental composition of the activated biocarbons.

Sample	C^daf^ [wt.%]	H^daf^ [wt.%]	N^daf^ [wt.%]	O^diff^ [wt.%]
AH_C	85.3	2.7	0.9	11.1
AH_M	71.9	2.6	1.3	24.2
AK_C	79.8	2.3	1.1	16.8
AK_M	78.2	2.2	1.1	18.5

^daf^—dry-ash-free basis; ^diff^—calculated by difference.

**Table 3:** Textural parameters of the activated biocarbons derived from sage stems.

Sample	Total ^1^		Micropore		Micropore Contribution [%]	Mean Pore Size [nm]
	Surface Area [m^2^/g]	Pore Volume [cm^3^/g]	Area [m^2^/g]	Volume [cm^3^/g]		
AH_C	820	0.837	116	0.045	5.4	4.08
AH_M	1151	1.103	224	0.093	8.4	3.84
AK_C	228	0.144	157	0.073	50.7	2.53
AK_M	471	0.255	376	0.174	68.2	2.17

^1^ method error in the range from 2 to 5%.

**Table 4:** The acid base properties of the activated biocarbons derived from sage stems.

Sample	pH of Water Extracts	Acidic Group Content [mmol/g]	Basic Group Content [mmol/g]	Total Content of Surface Groups [mmol/g]
Sage stems	6.37	1.55	1.25	2.80
AH_C	2.50	1.05	0.00	1.05
AH_M	2.32	2.76	0.00	2.76
AK_C	5.73	0.99	0.49	1.48
AK_M	5.45	1.08	0.33	1.41

**Table 5:** Langmuir and Freundlich parameters of the isotherms of methylene blue and Triton X-100 adsorption on the activated carbons derived from sage stems.

Sample	q_exp_ [mg/g]	Langmuir Model			Freundlich Model		
		q_m_ [mg/g]	R^2^	K_L_ [dm^3^/mg]	1/n	R^2^	K_F_ [mg/g (mg/dm^3^)^1/n^]
**Methylene Blue**							
Sage stems	5.9	6.2	0.985	0.15	0.305	0.904	2.65
AH_C	143.8	142.8	0.997	14.00	0.131	0.862	112.35
AH_M	289.6	285.7	0.999	35.00	0.085	0.980	257.22
AK_C	22.8	24.7	0.980	1.36	0.240	0.958	11.72
AK_M	56.3	55.9	0.999	29.83	0.069	0.964	49.70
**Triton X-100**							
AH_C	517.7	580.3	0.996	0.02	0.399	0.901	55.80
AH_M	644.3	708.1	0.996	0.03	0.303	0.879	116.32
AK_C	19.6	23.3	0.908	0.01	0.415	0.710	1.69
AK_M	38.8	54.7	0.970	0.01	0.452	0.886	3.12

**Table 6:** Kinetic parameters for methylene blue and Triton X-100 adsorption on the H_3_PO_4_-activated biocarbons derived from sage stems.

Sample	q_exp_ [mg/g]	Pseudo-First-Order			Pseudo-Second-Order		
		q_cal_ [mg/g]	k_1_ [1/min]	R^2^	q_cal_ [mg/g]	k_2_ [1/min]	R^2^
**Methylene Blue**							
AH_C	84.4	36.2	0.0135	0.897	86.0	0.0010	0.999
AH_M	187.9	19.0	0.0152	0.903	188.7	0.0023	0.999
**Triton X-100**							
AH_C	273.3	45.2	0.0221	0.924	275.2	0.0015	0.999
AH_M	361.9	31.5	0.0209	0.927	363.4	0.0020	0.999

**Table 7:** Stage parameters of the intraparticle diffusion kinetics model for Triton X-100 adsorption on the H_3_PO_4_-activated biocarbons derived from sage stems.

Sample	First Step		Second Step		Third Step	
	k_i1_ [mg/g min^0.5^]	R^2^_1_	k_i2_ [mg/g min^0.5^]	R^2^_2_	k_i3_ [mg/g min^0.5^]	R^2^_3_
**Methylene Blue**						
AH_C	3.134	0.992	1.778	0.975	0.872	0.977
AH_M	7.540	0.990	3.520	0.969	0.284	0.843
**Triton X-100**						
AH_C	16.088	0.997	3.073	0.781	0.265	0.663
AH_M	11.667	0.993	2.293	0.685	0.068	0.996

**Table 8:** Comparison of adsorption capacities of biomass-derived activated biocarbons towards organic pollutants (literature review).

Activated Biocarbon Precursor	Preparation Procedure	Maximal Adsorbed Amount [mg/g]	Reference
**Methylene Blue**			
Sage stems	Chemical activation with H_3_PO_4_ (conventional heating)	157.9	This study
Sage stems	Chemical activation with H_3_PO_4_ (microwave heating)	298.4	This study
Papaya seed, pineapple crown	Pyrolysis and functionalization with mixed acid solution (H_2_SO_4_ and H_3_PO_4_)	470.2	[21,22]
Pepper stalks	Chemical activation with H_3_PO_4_ (microwave heating)	75.0	[23]
Rubber seed pericarp	Chemical activation with H_3_PO_4_ (microwave heating)	347.8	[24]
Bamboo waste	Chemical activation with K_2_CO_3_ (microwave heating)	85.6	[25]
Almond shell	Chemical activation with ZnCl_2_ (microwave heating)	314.2	[26]
Sugarcane bagasse waste	Chemical activation with KOH (conventional heating)	136.5	[27]
**Triton X-100**			
Sage stems	Chemical activation with H_3_PO_4_ (conventional heating)	517.7	This study
Sage stems	Chemical activation with H_3_PO_4_ (microwave heating)	644.3	This study
Horsetail herb	Physical activation with steam (conventional heating)	20.0	[28]
Walnut shells	Chemical activation with H_3_PO_4_ (conventional heating)	61.8	[29]
Sweet cherry stones	Physical activation with carbon dioxide (microwave heating)	86.5	[30]

**Table 9:** Thermodynamic parameter values for the adsorption of methylene blue and Triton X-100 on H_3_PO_4_-activated biocarbons derived from sage stems at different temperatures.

Sample	T [°C]	T [K]	K_a_ [dm^3^/g]	ΔG^0^ [kJ/mol]	ΔH^0^ [kJ/mol]	ΔS^0^ [kJ/mol K]
**Methylene Blue**						
AH_C	20	293	14.93	−6.70	−17.22	−35.01
	30	303	13.28	−6.62		
	40	313	9.62	−5.99		
AH_M	20	293	43.55	−9.35	−30.81	−72.76
	30	303	22.05	−7.92		
	40	313	20.04	−7.93		
**Triton X-100**						
AH_C	20	293	8.94	−5.34	3.67	31.11
	30	303	10.79	−5.99		
	40	313	9.82	−5.94		
AH_M	20	293	15.55	−6.68	−2.65	13.54
	30	303	13.82	−6.62		
	40	313	14.53	−6.96		

## Data Availability

Data are contained within the article.
